# Natural polyphenol mangiferin delays neuronal cell senescence by inhibiting neuroinflammation mediated by microglial activation

**DOI:** 10.1016/j.ibneur.2025.04.006

**Published:** 2025-04-08

**Authors:** Wei Nong, Xiaoli Chen, Yixin Chen, Xueping Feng, Wen Kong, Rui Chi, Li Yan, Zhiquan Wei

**Affiliations:** a Guangxi Scientific Research Center of Traditional Chinese Medicine, Guangxi Traditional Chinese Medicine University, Nanning 530200, China; b Laboratory of Zhuang Medicine Prescriptions Basis and Application Research, Guangxi Traditional Chinese Medicine University, Nanning 530001, China; c Guangxi Key Laboratory of Efficacy Study on Chinese Materia Medica, Guangxi Traditional Chinese Medicine University, Nanning 530200, China; d School of Basic Medical Sciences, Youjiang Medical University for Nationalities, Baise 533000, China; e Zhuang Medicine College, Guangxi Traditional Chinese Medicine University, Nanning 530001, China

**Keywords:** Mangiferin, Microglial, AMPK-mTOR-IRF5 pathway, Alzheimer's disease, Neuroinflammation, Neuronal cell senescence

## Abstract

Extracellular β-amyloid protein (Aβ) plaques are prominent pathological feature of Alzheimer's disease (AD). Aβ oligomers and plaques induce sustained microglial activation via the adenosine monophosphate-activated protein kinase (AMPK)/mammalian target of rapamycin (mTOR)/interferon regulatory factor 5 (IRF5) signaling pathway. This microglial activation-mediated neuroinflammation can accelerate neuronal cell senescence. Consequently, the regulation of the AMPK/mTOR/IRF5 pathway presents a potential therapeutic target for AD, as it may inhibit neuroinflammation and delay neuronal cell senescence. Mangiferin, a bioactive natural polyphenol extracted from the leaves of *Mangifera indica Linn*., has garnered significant attention for its anti-inflammatory properties. However, it remains unclear whether mangiferin can modulate the AMPK/mTOR/IRF5 pathway to inhibit microglial activation-mediated neuroinflammation and delay neuronal cell senescence. This study employed both cellular and animal models of neuronal cell senescence to explore the effects of mangiferin on the regulation of the AMPK/mTOR/IRF5 pathway, aiming to inhibit neuroinflammation and delay neuronal cell senescence *in vitro* and *in vivo*. Specifically, SH-SY5Y neuroblastoma cells were subjected to a neuroinflammatory microenvironment induced by Aβ1–42-mediated HMC3 microglial activation to induce neuronal cell senescence *in vitro*. Additionally, SAMP8 accelerated aging mice were utilized as an aging animal model. The results indicate that mangiferin significantly enhances AMPK phosphorylation in microglial cells, inhibits mTOR activation, and downregulates IRF5 expression. These effects collectively suppress microglial activation and markedly reduce the production of pro-inflammatory cytokines by activated microglia. Consequently, there is a decrease in the proportion of neurons arrested in the G0/G1 phase and a reduction in the number of senescence-associated β-galactosidase (SA-β-gal) positive neurons. Furthermore, mangiferin significantly decreases the expression of neuronal cell senescence markers P16Ink4a and P21Cip1. Collectively, these findings suggest that mangiferin effectively regulates the AMPK/mTOR/IRF5 pathway, inhibits neuroinflammation mediated by microglial activation, and delays neuronal cell senescence. This study underscores the potential of mangiferin for the treatment of neuroinflammation and neurodegenerative diseases.

## Graphical Abstract

**Figure:**
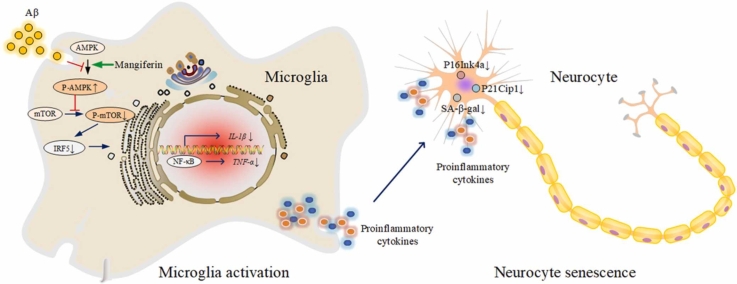

## 1 Introduction

Alzheimer's disease (AD) is a prevalent neurodegenerative disorder characterized by progressive cognitive decline and memory loss. Projections indicate that by 2030 and 2050, the number of individuals affected by AD will reach 82 million and 152 million, respectively. This increasing incidence of AD is evident in both developed and developing nations, presenting a significant public health challenge and imposing a considerable socioeconomic burden (Tahami Monfared et al., 2022).

A hallmark pathological feature of AD is the abnormal accumulation of extracellular β-amyloid protein (Aβ), which forms oligomers and plaques that contribute to cognitive and memory impairment in affected patients (Zaletel et al., 2021). Microglia, the primary immune cells in the central nervous system, play a critical role in the pathogenesis of AD. The presence of Aβ oligomers and plaques leads to persistent activation of pattern recognition receptors and related complements on the surface of microglia, resulting in the release of various inflammatory mediators and neurotoxic substances that create a neuroinflammatory microenvironment (Salminen et al., 2009). Due to their high metabolic demands and limited regenerative capacity, neurons are particularly susceptible to direct damage from inflammation (Hatami et al., 2019, Heneka et al., 2013). The sustained activation of microglia within this neuroinflammatory microenvironment can accelerate neuronal cell senescence, a process referred to as inflammaging (Singh et al., 2014). Accumulating evidence suggests that aged neurons not only lose their normal function but also adopt a pronounced pro-inflammatory phenotype, producing excessive amounts of inflammatory mediators. Consequently, the interplay between neuronal cell senescence and neuroinflammation can establish a mutually reinforcing pathological cycle (Dhapola et al., 2021). It is clear that Aβ-induced microglial activation is instrumental in perpetuating neuroinflammation and accelerating neuronal cell senescence.

Recent studies have indicated that dysregulation of the adenosine monophosphate-activated protein kinase (AMPK)/mammalian target of rapamycin (mTOR)/interferon regulatory factor 5 (IRF5) signaling pathway is implicated in neuroinflammation and the pathogenesis of AD (Zhu et al., 2016, Li et al., 2020). Notably, the upregulation of IRF5 expression has been shown to promote microglial activation (Zhao et al., 2017). Furthermore, AMPK activation can inhibit mTOR phosphorylation, leading to downregulation of IRF5 expression, suppression of microglial activation, and subsequent alleviation of neuroinflammation (Kim et al., 2023). Consequently, the AMPK/mTOR/IRF5 pathway is critically involved in the regulation of neuroinflammation and neuronal cell senescence (Qin et al., 2020). By activating AMPK in microglia, it is possible to inhibit mTOR activation, reduce IRF5 expression, and ultimately prevent microglial activation. This approach may serve to mitigate neuroinflammation and neuronal cell senescence mediated by microglial activation (Muzammil et al., 2021, Ngwa et al., 2022).

**Fig. 1:**
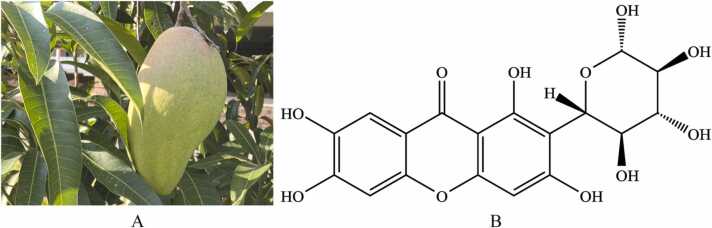
Leaves of *Mangifera indica Linn*. (A) and the chemical structure of mangiferin (B).

Leaves of *Mangifera indica Linn*. (Fig. 1A) have been employed as traditional herbal medicine in southern China for an extended period. For centuries, mango leaves, either alone or in conjunction with other therapeutic agents, have been utilized to treat inflammatory diseases (Kumar et al., 2021). The primary active compound in mango leaves, mangiferin, is a natural polyphenol (Fig. 1B) that has attracted considerable attention due to its remarkable anti-inflammatory properties and health benefits (Lei et al., 2021, Li et al., 2021). Existing research indicates that certain natural polyphenols can activate AMPK (Sadek et al., 2024, Li et al., 2022). Mangiferin has been shown to downregulate the expression of IRF5 in macrophages, thereby inhibiting their activation (Wei et al., 2016). Given that microglia exhibit many biological similarities to macrophages (Blecharz-Lang et al., 2022, Narvaiz et al., 2022), it is pertinent to explore whether mangiferin can inhibit microglial activation by modulating the AMPK/mTOR/IRF5 pathway, potentially alleviating neuroinflammation and delaying neuronal cell senescence. Consequently, we investigated the effects of mangiferin on microglial activation-mediated neuroinflammation and neuronal cell senescence both *in vivo* and *in vitro*. This study offers new insights into the potential role of mangiferin in addressing neuroinflammation and neurodegenerative diseases.

## 2 Materials and methods

### 2.1 Key reagents

Mangiferin was purchased from Aladdin (Shanghai, CN). β-amyloid 1–42 (Aβ1–42) and dimethyl sulfoxide (DMSO) were obtained from Sigma Aldrich (St. Louis, MO, USA), while ibuprofen was obtained from Johnson & Johnson (New Brunswick, NJ, USA). The mTOR activator MHY1485 was purchased from Selleck (Houston, TX, USA). The anti-P16Ink4a antibody, anti-P21Cip1 antibody, and diaminobenzidine (DAB) chromogenic solution were obtained from Solarbio (Beijing, CN). The DMEM cell culture medium, activated charcoal-absorbed fetal bovine serum (FBS) and Accutase collagenase cell dissociation solution were obtained from Gibco (Grand Island, NY, USA) and eBioscience (San Diego, CA, USA), respectively. The CCK-8 cell proliferation activity detection kit was obtained from Dojindo (Mashiki, Kamimashiki, JP). The enzyme-linked immunosorbent assay (ELISA) kits for human interleukin-1β (IL-1β) and tumor necrosis factor α (TNF-α) were purchased from Cusabio (Wuhan, Hubei, CN). SurePAGE protein electrophoresis precast gel was obtained from Genscript (Nanjing, Jiangsu, CN). The senescence-associated β-galactosidase (SA-β-gal) staining kit, cell cycle and apoptosis detection kit, Western cell lysis buffer, BCA protein concentration determination kit, ECL chemiluminescence detection kit, phosphate-buffered saline (PBS), Alexa Fluor 647-conjugated goat anti-rabbit IgG, and 4,6-diamino-2-phenyl indole (DAPI) staining solution were purchased from Beyotime (Shanghai, CN). BlockAid blocking solution, Image iT fixation/permeabilization kit, and ActinGreen 488 ReadyProbes were obtained from Invitrogen (Carlsbad, CA, USA). Anti-AMPK alpha 1 + AMPK alpha 2 antibody, anti-AMPK alpha 1 (phospho T183) + AMPK alpha 2 (phospho T172) antibody, anti-mTOR antibody, anti-mTOR (phospho S2448) antibody, anti-IRF5 antibody, and anti-glyceraldehyde-3-phosphate dehydrogenase (GAPDH) antibody were purchased from Abcam (Boston, MA, USA). Additionally, anti-mTOR (phospho-Ser2448) antibody, anti-IL-1β antibody, anti-TNF-α antibody, and horseradish peroxidase (HRP)-conjugated goat anti-rabbit IgG were obtained from Sangon (Shanghai, CN).

### 2.2 Primary instruments

The primary instruments utilized in this study were the Mini-PROTEAN vertical electrophoresis system, Mini Trans-blot transfer system, and ChemiDoc imaging system (Bio-Rad, Hercules, CA, USA). Additionally, the 5430 R refrigerated centrifuge (Eppendorf, Wesseling-Berzdorf, DE), Infinite 200 Pro microplate reader (Tecan, Männedorf, CH), DMi8 research-grade microscope, ASP300S fully automated tissue dehydrator, RM2245 semi-automatic rotary microtome, and EG1150H paraffin embedding system (Leica, Wetzlar, DE) were used. Finally, the LSR Fortessa multi-color flow cytometer (Becton Dickinson, Franklin Lakes, NJ, USA) was utilized.

### 2.3 Cell culture

The HMC3 human microglial cell line and the SH-SY5Y human neuroblastoma cell line were obtained from Procell (Wuhan, Hubei, China). Both cell lines were cultured in DMEM supplemented with 10 % FBS, 100 IU/mL penicillin, and 100 mg/mL streptomycin. The initial cell density was maintained at 2 × 10^4^ cells/cm^2^. Cultivation was conducted in a CO_2_ incubator set at 37°C with 5 % CO_2_ and saturated humidity. The passage cycle was established at approximately 4 d, thereby ensuring that the cells remained healthy and viable throughout the experiment.

### 2.4 Evaluation of the toxicity of mangiferin to HMC3 cells

To assess the toxicity of mangiferin to HMC3 cells, cells in the logarithmic growth phase were seeded in cell culture plates at an initial density of 2 × 10^4^ cells/cm^2^. Mangiferin was prepared by dissolving it in DMEM supplemented with 0.1 % DMSO. A control group without drug treatment, along with treatment groups at varying concentrations of mangiferin (12.5, 25, 50, 100, 200, 300, 400, 500, 600, and 700 μM), was established, with three replicates for each concentration. The plates were incubated in a cell culture incubator for 24 h, after which 10 μL of CCK8 solution was added and the incubation continued for an additional 2 h. The culture supernatant was then collected, and the optical absorbance was measured at 450 nm using an ELISA reader. The safe concentration of mangiferin for subsequent experiments was determined based on the results of the cell toxicity test.

### 2.5 Establishment of an in vitro cellular model of neuronal cell senescence in an inflammatory microenvironment

The experiment is divided into two steps, prioritised as follows: (1) Activation of HMC3 cells: HMC3 cells in the logarithmic growth phase were seeded at an initial cell density of 2 × 10^4^ cells/cm^2^ in a cell culture dish. Aβ1–42 was added at a final concentration of 20 μM, and the cells were activated for 72 h(Wan et al., 2021; Ma et al., 2022). The medium was then replaced with fresh DMEM, and HMC3 cells were further cultured. (2) Co-culture of SH-SY5Y cells with activated HMC3 cells: SH-SY5Y cells cultured alone served as the control group. A co-culture model of SH-SY5Y cells with activated HMC3 cells was established using Transwell cell culture inserts (Corning, Corning, NY, USA). Activated HMC3 cells were placed in the upper chamber, and SH-SY5Y cells were placed in the lower chamber. Both cell types were co-cultured for 72 h. The cell culture supernatant was collected, and the concentrations of inflammatory markers IL-1β and TNF-α were measured using ELISA to verify the inflammatory microenvironment. SH-SY5Y cells in the lower chamber were collected, and the expression levels of the senescence markers P16Ink4a and P21Cip1 were measured by Western blotting to verify neuronal cell senescence.

### 2.6 Effects of mangiferin on microglial activation induced by Aβ1–42

HMC3 cells in the logarithmic growth phase were initially seeded at a density of 2 × 10^4^ cells/cm^2^ in cell culture dishes. The experimental groups included a normal control group, a model control group, and mangiferin treatment groups at varying concentrations, respectively pre-incubated with mangiferin at final concentrations of 12.5, 25, and 50 μM for 24 h. Additionally, there was a group that received both the mTOR activator MHY1485 and mangiferin, where cells were pre-incubated with 10 μM MHY1485 for 6 h (Wang et al., 2021), then incubated with mangiferin at a final concentration of 50 μM for 24 h. Subsequently, after replacing the culture medium with fresh medium, all groups, except for the normal control group, were treated with Aβ1–42 at a final concentration of 20 μM for 72 h. The follow-up procedures included the following steps: (1) The cell culture supernatant was collected, and the concentrations of the inflammatory markers IL-1β and TNF-α were measured using ELISA. (2) HMC3 cells were harvested, and the expression levels of AMPKα1 +AMPKα2, phospho-(AMPKα1 +AMPKα2), mTOR, phospho-mTOR (S2448), and IRF5 were assessed using Western blotting. (3) The in-situ expression of IRF5 in HMC3 cells was demonstrated by using a cellular immunofluorescence staining.

### 2.7 Indirect effects of mangiferin on neuronal cell senescence in neuroinflammatory microenvironment

6 experimental groups were set up, including the normal control group (SH-SY5Y cells cultured alone), the model control group, and the groups treated with different concentrations of mangiferin and MHY1485 co-treated with mangiferin. The different mangiferin treatment group consisted of 3 subgroups, in which HMC3 cells were pre-incubated with 12.5, 25 or 50 μM mangiferin for 24 h. HMC3 cells in the MHY1485 co-treatment group were pre-incubated with 10 μM MHY1485 for 6 h, followed by 50 μM mangiferin for 24 h. Afterwards, with the exception of the normal control group, the method described in Section 2.5 was employed to activate the HMC3 cells in the remaining 5 subgroups. Thereafter, SH-SY5Y cells were co-cultured with HMC3 cells for 72 h using Transwell cell culture inserts, with the objective of inducing senescence in the SH-SY5Y cells. Subsequent procedures included: (1) collecting SH-SY5Y cells to detect the expression levels of aging markers P16Ink4a and P21Cip1 via Western blotting; (2) analyzing the percentage of SH-SY5Y cells in the G0/G1 phase using flow cytometry; and (3) staining SH-SY5Y cells to observe the aging marker SA-β-gal under a microscope.

### 2.8 Impacts of mangiferin on neuroinflammation and neuronal cell senescence in SAMP8 accelerated aging mice

The experimental protocol employed in this study underwent thorough examination and received approval from the Ethics Committee of Guangxi University of Traditional Chinese Medicine (approval No. DW20211210–204). A total of 8 male, 8-week-old SPF SAMR1 normal aging mice and 40 male, 8-week-old SPF SAMP8 accelerated aging mice, each weighing between 18 and 22 g, were procured from the Experimental Animal Science Department of Peking University School of Medicine in China. All mice were housed in sanitized animal rooms maintained at a temperature of 20 ± 2°C and a relative humidity of 55 ± 15 %. The SAMP8 accelerated aging mice were randomly assigned to one of the following groups: a model group receiving distilled water (1 mL/d via gavage), an ibuprofen group (30 mg/kg/d via gavage) (Fielder et al., 2020, Liu et al., 2020), and low-, medium-, and high-dose mangiferin groups (25, 50, and 100 mg/kg/d via gavage) (Davis, 2002, Koenigsknecht and Landreth, 2004), with 8 mice in each group. The treatment duration was 4 weeks. At the conclusion of the 4 weeks, the mice were anesthetized via intraperitoneal injection of 2 % sodium pentobarbital (40 mg/kg), and their brains were placed on a cold operation table maintained at 4°C following craniotomy. A small sample of temporal lobe brain tissue was collected for Western blotting analysis to assess the expression levels of AMPKα1 +AMPKα2, phospho-(AMPKα1 +AMPKα2), mTOR, phospho-mTOR (S2448), IRF5, IL-1β, TNF-α, P16Ink4a, and P21Cip1. The remaining brain tissues were fixed in 10 % neutral formaldehyde, paraffin sections were prepared for immunohistochemical staining of the aging markers P16Ink4a and P21Cip1.

### 2.9 ELISA detection of proinflammatory cytokines in cell culture supernatants

Cell culture supernatants were stored at −80°C and were fully thawed at 4°C prior to ELISA detection. Following the manufacturer's instructions, the samples were gently mixed before proceeding with the ELISA detection.

### 2.10 Protein Expression Measurement by Western Blotting

Cells or minced tissue samples were washed twice with PBS and subsequently resuspended in lysis buffer at a volume ten times greater than the sample volume. Following a 20-minute incubation at 4°C, the samples were centrifuged at 12,000 rpm for 5 min. The supernatant was collected for protein quantification. Equal amounts of protein (40 μg) were loaded onto precast protein gels for vertical electrophoresis. After electrophoresis, a wet transfer was conducted, and the membranes were incubated overnight at 4°C with the primary antibody. The dilution ratios for GAPDH, IL-1β, and TNF-α primary antibodies were set at 1:1200, while other primary antibodies were diluted at 1:1000. Following the incubation with primary antibodies, the membranes were treated with secondary antibodies diluted to 1:2000 at room temperature for 60 min. Subsequently, the membranes were incubated with ECL chemiluminescence solution for 3 min, and images were captured using the ChemiDoc imaging system. The relative expression levels of target proteins were determined by comparing the band intensities of the target proteins to those of the internal reference protein, GAPDH.

### 2.11 Analysis of the cell cycle by flow cytometry

SH-SY5Y cells were harvested, and the culture medium was subsequently removed. The cells were washed twice with PBS and digested with collagenase to obtain individual cells. Following digestion, the cells were incubated in 70 % ethanol at 4°C overnight. After the ethanol was removed, the cells were washed twice with PBS at 4°C and then incubated in a cell cycle staining solution at 37°C in the dark for 30 min. The cell cycle was detected and analyzed using the LSR Fortessa flow cytometer.

### 2.12 Detection of neuronal cell senescence by SA-β-gal staining

SH-SY5Y cells were harvested, and the culture medium was discarded. The cells were washed twice with PBS and subsequently fixed in a fixative solution at room temperature for 15 min. Following fixation, the staining solution was added, and the cells were incubated overnight at 37°C. After the incubation period, the staining solution was removed, and an appropriate volume of PBS was added. The cells were then examined under a conventional optical microscope to assess SA-β-gal staining.

### 2.13 Immunofluorescence analysis of in situ expression of IRF5 in HMC3 cells

HMC3 cells were harvested, and the culture medium was subsequently removed. The cells were washed twice with PBS, followed by fixation and permeabilization. An anti-IRF5 antibody, diluted to 1:200, was added and incubated in the dark at 4°C overnight. Following the removal of the primary antibody, the cells were washed twice with PBS and incubated in the dark with Alexa Fluor 647-conjugated goat anti-rabbit IgG, diluted to 1:500, at 25°C for 2 h. After the secondary antibody was removed, the cells were washed twice with PBS and incubated with ActinGreen 488 ReadyProbes at room temperature in the dark for 60 min. Finally, the cells were stained with DAPI at room temperature in the dark for 5 min. After the staining solution was removed, an appropriate volume of PBS was added, and the cells were observed for fluorescence using a DMi8 fluorescence microscope.

### 2.14 Immunohistochemical staining of brain tissue

Mouse brain tissue, fixed in 10 % neutral formaldehyde, was utilized to prepare routine paraffin sections with a thickness of 20 μm. The sections underwent deparaffinization and hydration, followed by incubation at room temperature for 3 h with primary antibodies against P16Ink4a or P21Cip1, each diluted to 1:200. Subsequently, the sections were incubated with HRP-conjugated secondary antibodies, diluted to 1:500, at room temperature for 2 h, and then subjected to DAB staining. Finally, the sections were counterstained with hematoxylin and examined under a conventional optical microscope, allowing for the calculation of average optical density values of positive staining.

### 2.15 Statistical analysis

Data were normalized to mean ± standard deviation ($\bar{x}\pm s$) and were found to conform to a normal distribution with homogeneity of variance. Statistical analyses were conducted using SPSS version 22.0 (IBM, Armonk, NY, USA). Additionally, SigmaPlot version 12.5 (Systat Software, San Jose, CA, USA) was employed for graphing and visualizing the results. Independent sample two-tailed t-tests were performed to assess differences between the two groups, while one-way analysis of variance (ANOVA) was utilized to compare differences among multiple groups. For post hoc analysis, Tukey's multiple comparison test was applied. *P*-values were derived from two-tailed tests, with a significance threshold set at *P* < 0.05.

## 3 Results

### 3.1 Evaluation of the toxicity of mangiferin to HMC3 cells

**Fig. 2:**
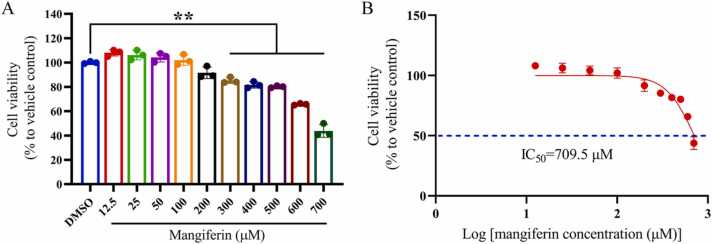
Evaluation of the toxicity of mangiferin to HMC3 cells. Each group contained 3 independent samples and data were expressed as $\bar{x}\pm s$ and analyzed by one-way ANOVA. ^**^P < 0.01, compared with DMSO group as control.

The results indicated that, in comparison to the control group, mangiferin at lower concentrations (12.5–200 μM) did not demonstrate significant cytotoxicity. Conversely, at higher concentrations (300–700 μM), mangiferin exhibited cytotoxic effects (Fig. 2A). Utilizing the calculation method outlined by Sebaugh et al. (Sebaugh, 2011), the median inhibitory concentration (IC50) of mangiferin on HMC cells was determined to be 709.5 μM (Fig. 2B). These findings imply that mangiferin is safe for HMC3 cells at lower concentrations, but may negatively impact cell viability at elevated concentrations. Consequently, subsequent cytopharmacological experiments were performed using safe and lower concentrations of mangiferin.

### 3.2 Establishment of an in vitro cellular model of neuronal cell senescence in the inflammatory microenvironment

**Fig. 3:**
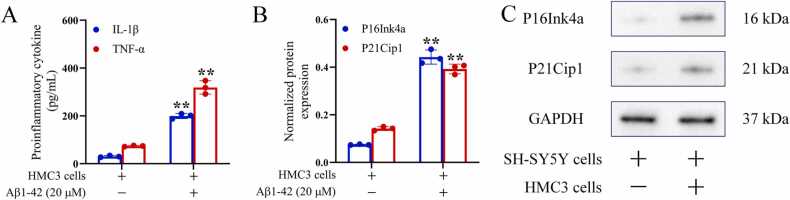
Establishment of an *in vitro* cellular model of neuronal cell senescence in the inflammatory microenvironment. The levels of IL-1β and TNF-α in the cell culture supernatants of each group are shown in Fig. 3A. The expression levels of senescence markers P16Ink4a and P21Cip1 in SH-SY5Y cells of each group are shown in Fig. 3B. Western blotting of P16Ink4a and P21Cip1 in SH-SY5Y cells is shown in Fig. 3C. Each group contained 3 independent samples, and data were expressed as $\bar{x}\pm s$ with independent samples *t*-test. ^**^P < 0.01, compared with the unstimulated HMC3 cells.

In the absence of Aβ1–42-induced activation treatment, IL-1β (30.43 ± 3.68 pg/mL) and TNF-α (73.99 ± 2.44 pg/mL) were found to be present at low levels in HMC3 cell culture supernatants. Conversely, HMC3 cells exposed to Aβ1–42-induced activation exhibited a substantial release of pro-inflammatory cytokines, resulting in a pronounced increase in the levels of IL-1β (199.28 ± 10.74 pg/mL) and TNF-α (318.81 ± 27.75 pg/mL) in the cell culture medium, which were significantly higher than those in HMC cells that did not receive Aβ1–42 stimulation (*P* < 0.01), suggesting that Aβ1–42 stimulation prompted HMC3 cells to differentiate towards a pro-inflammatory phenotype (Fig. 3A). Following co-culturing of activated HMC3 cells with SH-SY5Y cells, the expression levels of cellular senescence markers P16Ink4a (0.44 ± 0.03) and P21Cip1 (0.39 ± 0.02) in SH-SY5Y cells were significantly higher than those of SH-SY5Y cells cultured alone (*P* < 0. 01), which were significantly higher than those of P16Ink4a (0.44 ± 0.03) and P21Cip1 (0.39 ± 0.02) expression levels (*P* < 0.01), suggesting that the presence of the inflammatory microenvironment significantly accelerated cellular senescence in SH-SY5Y cells (Fig. 3B and C).

### 3.3 Inhibition of mangiferin on Aβ1–42-induced activation of microglia *in vitro*

**Fig. 4:**
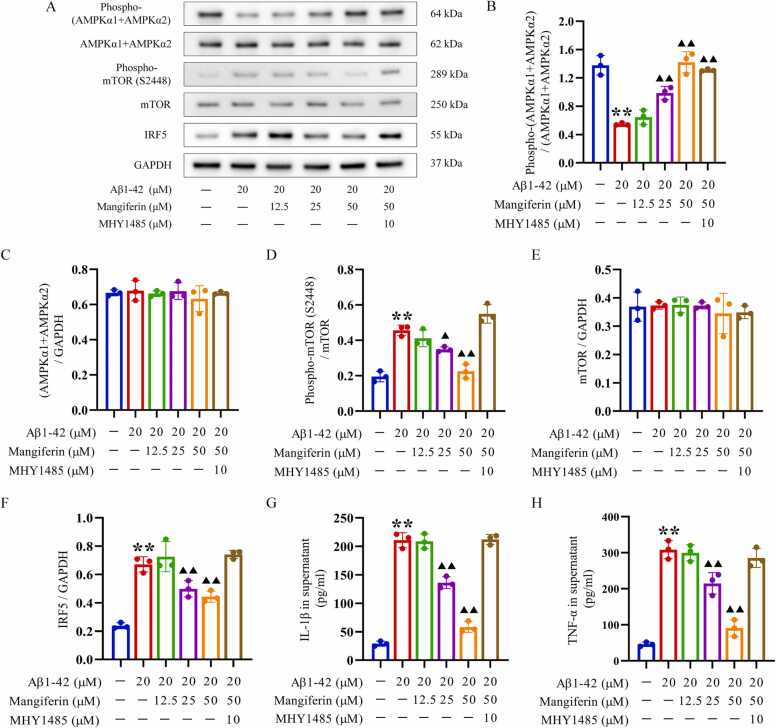
Inhibition of mangiferin on Aβ1–42-induced activation of microglia *in vitro*. Panel A shows Western blotting of key proteins in the AMPK/mTOR/IRF5 pathway (including AMPK, mTOR, their phosphorylated forms and IRF5) in HMC3 cells. Panels B-F show the levels of non-phosphorylated/phosphorylated AMPK, mTOR and IRF5 in HMC3 cells treated with different concentrations of mangiferin or the mTOR activator MHY1485. Panels G and H show the levels of IL-1β and TNF-α in the culture supernatant of HMC3 cells. Each group contained 3 independent samples and data were expressed as $\bar{x}\pm s$ deviation with one-way ANOVA and Tukey follow-up test. ^**^P < 0.01, compared with the unstimulated HMC3 cells. ▴ *P* < 0.05 and ▴▴ *P* < 0.01, compared with the activated HMC3 cells without drug treatment.

Immunoblotting (Fig. 4A) and ELISA detection of each cell group showed that not only did the IL-1β (210.82 ± 13.13 pg/mL) and TNF-α levels (308.44 ± 25.36 pg/mL) levels in the cell culture supernatant were significantly increased (Fig. 4G and H), but also intracellular Phospho-(AMPKα1 +AMPKα2) (0.37 ± 0.04) was decreased after stimulation with Aβ1–42 (Fig. 4B). However, intracellular phospho-mTOR (S2448) (0.17 ± 0.01) and IRF5 (0.67 ± 0.05) levels were significantly elevated (Fig. 4D and F). Compared with IL-1β (29.06 ± 4.45 pg/mL), TNF-α (45.77 ± 5.94 pg/mL) and intracellular Phospho-(AMPKα1 +AMPKα2) (0.92 ± 0.08), phospho-mTOR (S2448) (0.07 ± 0.00), IRF5 (0.24 ± 0.02) levels in cell culture supernatants of HMC3 cells not stimulated by Aβ1–42 (Fig. 4B, D, F, G, H), these differences were statistically significant (*P* < 0.01). Intracellular phospho-mTOR (S2448) (0.13 ± 0.00 and 0.08 ± 0.01, respectively), IRF5 (0.50 ± 0.06 and 0.44 ± 0.04, respectively) levels and IL-1β(136.08 ± 10.44 and 58.1 ± 60.01, respectively), TNF-α levels (214.67 ± 29.87 and 90.80 ± 23.06 pg/mL, respectively) levels in cell culture supernatants were significantly lower than those of HMC3 cells stimulated with Aβ1–42 alone (Fig. 4D, F, G, H) and had higher intracellular Phospho-(AMPKα1 +AMPKα2) (0.66 ± 0.02 and 0.89 ± 0.09, respectively) levels (Fig. 4B). These differences were statistically significant compared to HMC3 cells stimulated with Aβ1–42 alone (*P* < 0.05 or 0.01). However, 12.5 μM mangiferin did not cause similar changes.

Notably, when HMC3 cells were treated with the mTOR activator MHY1485 prior to mangiferin treatment, even though 50 μM of mangiferin significantly increased intracellular Phospho-(AMPKα1 +AMPKα2) (0.87 ± 0.02) (Fig. 4B), it did not prevent the emergence of higher intracellular levels of phospho-mTOR (S2448) (0.19 ± 0.01) and IRF5 (0.74 ± 0.03) (Fig. 4D, F). High levels of IL-1β (212.15 ± 8.65 pg/mL) and TNF-α (285.50 ± 26.48 pg/mL) remained in the cell culture supernatant. Immunofluorescence staining to assess in situ expression of IRF5 in different cell groups also corroborated the immunoblotting results. These results suggest that higher concentrations of mangiferin can antagonize the activation of HMC3 cells induced by Aβ1–42 stimulation, which is closely related to the AMPK-mTOR-IRF5 pathway.

**Fig. 5:**
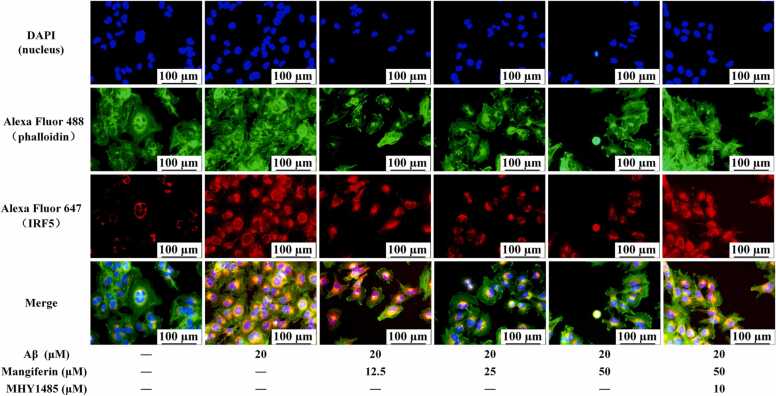
In-situ expression of IRF5 in HMC3 cells detected by immunofluorescence staining. Cell nuclei were stained blue using DAPI, while the cell cytoskeleton was stained green with phalloidin labeled with Alexa Fluor 488. The transcription factor IRF5 was subsequently stained red, first with an IRF5 primary antibody, followed by a secondary antibody labeled with Alexa Fluor 647. Scale bar is 100 μm.

### 3.4 Indirect delaying effect of mangiferin on neuronal cell senescence in the *in vitro* simulated inflammatory microenvironment

**Fig. 6:**
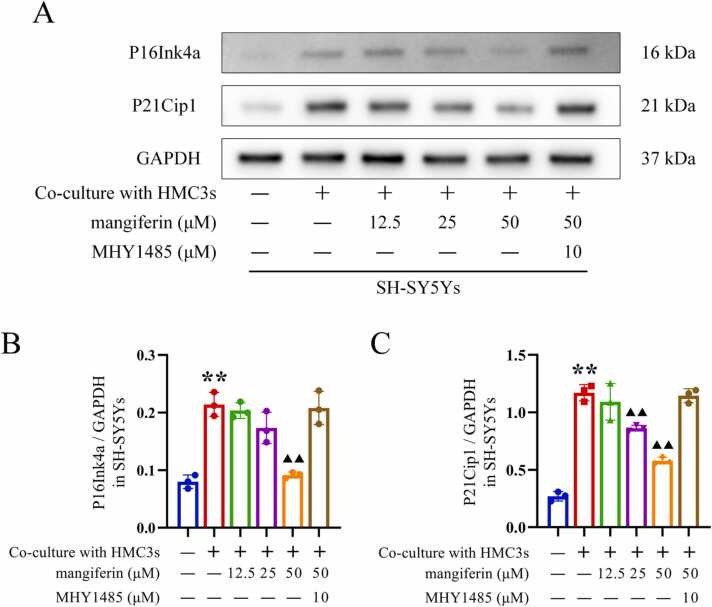
Effect of mangiferin on P16Ink4a and P21Cip1 protein expression levels in SH-SY5Y cells in the microenvironment of HMC3 cell activation-mediated inflammation. Panel A shows Western blotting of P16Ink4a and P21Cip1 proteins in SH-SY5Y cells. Panels B and C present statistical bar graphs illustrating the protein expression levels of P16Ink4a and P21Cip1 in SH-SY5Y cells within each group. Each group contained 3 independent samples and data were expressed as $\bar{x}\pm s$ deviation with one-way ANOVA and Tukey follow-up test. ^**^P < 0.01, compared with the SH-SY5Y cells cultured alone. ▴ *P* < 0.05 and ▴▴ *P* < 0.01, compared to the SH-SY5Y cells co-cultured with activated HMC3 cells and without drug treatment.

**Fig. 7:**
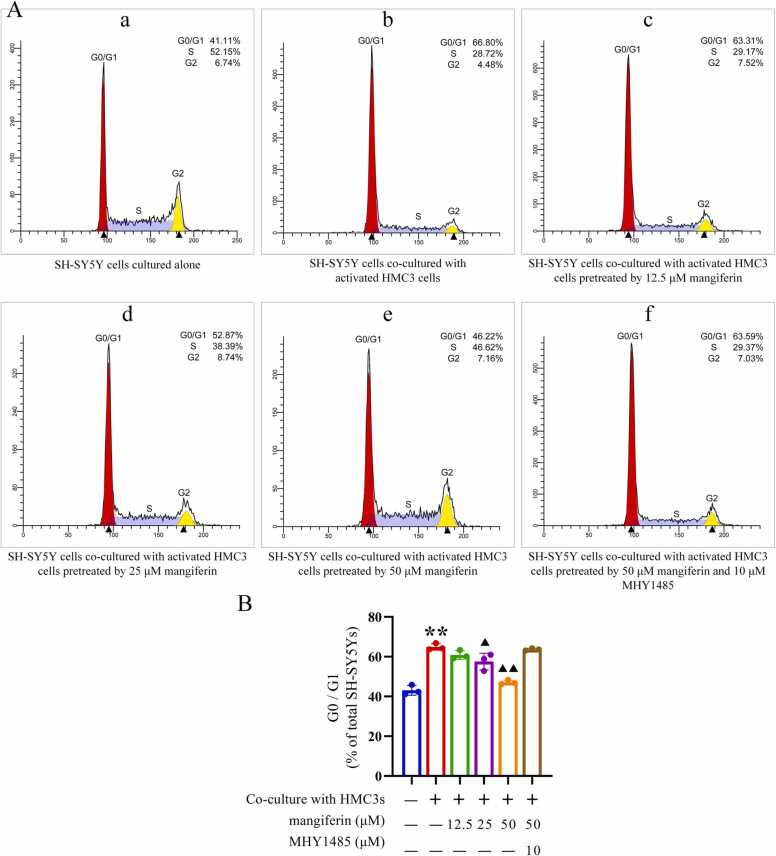
Effect of mangiferin on the cell cycle of SH-SY5Y cells in the inflammatory microenvironment mediated by HMC3 cell activation. As depicted in Panel A, the flow cytometry histograms illustrate the percentage of SH-SY5Y cells from each group that have halted in the G0/G1 phase. Panel B presents a statistical bar chart that illustrates the results of the statistical comparison of the cell cycle data for each group. Each group contained 3 independent samples and data were expressed as $\bar{x}\pm s$ deviation with one-way ANOVA and Tukey follow-up test. ^**^P < 0.01, compared with the SH-SY5Y cells cultured alone. ▴ *P* < 0.05 and ▴▴ *P* < 0.01, compared to the SH-SY5Y cells co-cultured with activated HMC3 cells and without drug treatment.

**Fig. 8:**
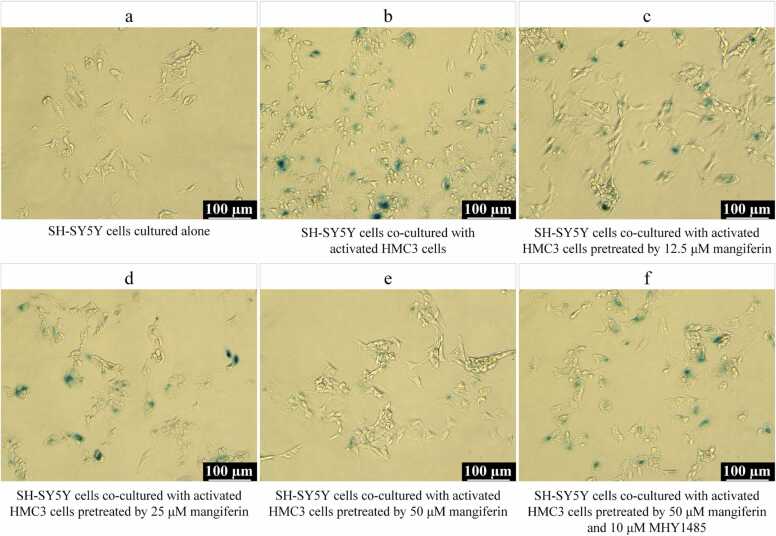
SA-β-gal staining of SH-SY5Y cells in the microenvironment of HMC3 cell activation-mediated inflammation. Localized areas of senescent cells were stained dark blue. Scale bar is 100 μm.

In the inflammatory microenvironment caused by Aβ1–42-induced activation of HMC3 cells, SH-SY5Y cells exhibited significantly increased levels of P16Ink4a (0.21 ± 0.02) and P21Cip1 (1.17 ± 0.07), and the proportion of cells retarded in the G0/G1 phase [(64.90 ± 1.67) %] also increased significantly, and the number of senescent SH-SY5Y cells was significantly higher by SA-β-gal staining. These senescence-related indexes were found to be significantly higher than the levels of P16Ink4a (0.08 ± 0.01), P21Cip1 (0.27 ± 0.04) and the proportion of cells in G0/G1 phase [(43.10 ± 2.49) %] of SH-SY5Y cells cultured alone, and the difference between them was statistically significant (*P* < 0.01). When HMC3 cells were pretreated with 25 or 50 μM mangiferin and then activated by Aβ1–42, SH-SY5Y cells in the inflammatory microenvironment caused by these HMC3 cells showed only relatively few levels of P16Ink4a (0.17 ± 0.03 and 0.09 ± 0.01, respectively), P21Cip1 (0.86 ± 0.02 and 0.58 ± 0.03, respectively) and the proportion of cells arrested in the G0/G1 phase [(57.56 ± 4.19) % and (47.06 ± 1.05)%], respectively, SA-β-gal staining showed fewer senescent SH-SY5Y cells, which were lower than SH-SY5Y cells in the inflammatory microenvironment caused by HMC3 cells not pretreated with mangiferin (*P* < 0.05 or 0.01). However, 12.5 μM mangiferin pretreatment failed to result in similar improvements in aging-related markers. These results (shown in Fig. 6, Fig. 7, Fig. 8) indicate that the accelerated senescence of SH-SY5Y cells within the neuroinflammatory microenvironment was also improved after higher concentrations of mangiferin inhibited the activation of HMC3 cells induced by Aβ1–42. This is also indicative of the close association among microglia, neuroinflammatory microenvironment and neuronal cell senescence.

While HMC3 being subjected to treatment with mTOR activator MHY1485, followed by the administration of 50 μM mangiferin, and subsequent activation of HMC3 cells induced by Aβ1–42, SH-SY5Y cells in the inflammatory microenvironment caused by these HMC3 cells still exhibited a notable elevation in the levels of P16Ink4a (0.21 ± 0.03) and P21Cip1 (1.15 ± 0.06), and the proportion of cells that were retarded in the G0/G1 phase [(63.62 ± 0.58)%) SA-β-gal staining showed that senescent SH-SY5Y cells were still abundant, and these indicators were not statistically different from SH-SY5Y cells in the inflammatory microenvironment caused by HMC3 cells not pretreated with mangiferin. As illustrated in Fig. 6, Fig. 7, Fig. 8, this finding indicates that the inhibition of intracellular mTOR activation of HMC3 plays a pivotal role in mangiferin inhibiting neuroinflammation.

It is important to note that, in contrast to the proliferative capacity exhibited by SH-SY5Y cells, human neural cells, as mature differentiated cells, do not demonstrate a comparable level of proliferation. Consequently, the potential pharmacotherapeutic significance of the cell cycle assay results should be interpreted with caution. However, it is now well established that human neuronal cells can still possess varying degrees of proliferative differentiation capacity under certain pathological conditions. Consequently, neuroregeneration research has been a continuing hot topic for many years. Therefore, the neuroprotective effects of mangiferin in such cases should not be overlooked.

### 3.5 Inhibition of mangiferin on neuroinflammation and neuronal cell senescence in SAMP8 accelerated aging mice via the modulation of the AMPK/mTOR/IRF5 pathway

**Fig. 9:**
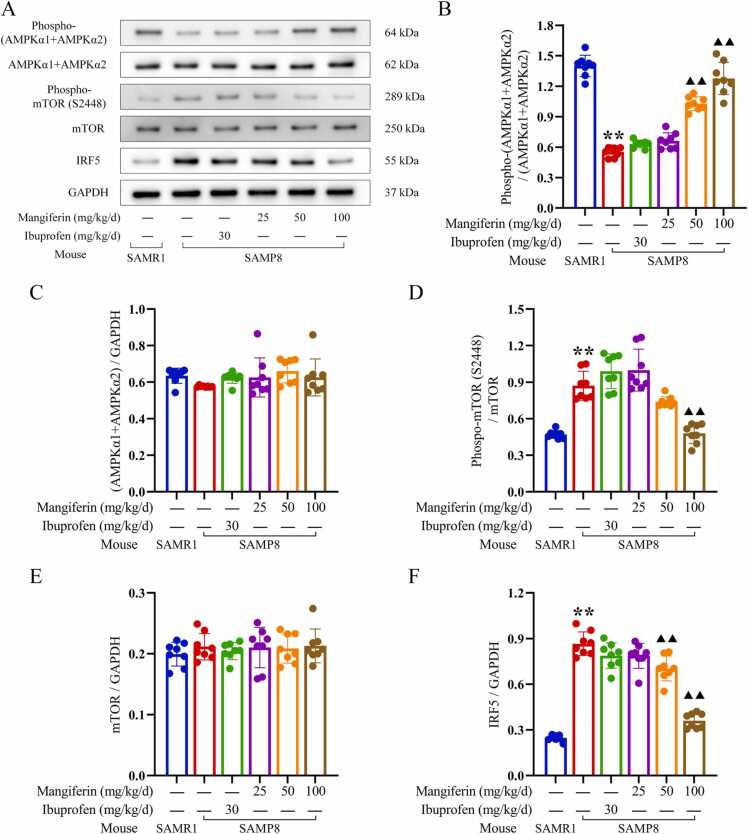
Regulation of the AMPK/mTOR/IRF5 pathway by mangiferin in the temporal lobe brain tissue of the cerebral cortex of SAMP8-accelerated aging mice. 8-week-old normal aging SAMR1 mice were used as the normal control group. The 8-week-old SAMP8 mice in the remaining drug intervention groups were treated with ibuprofen or different doses of mangiferin for a period of 4 weeks. Panel A presents the results of Western blots of key proteins of the AMPK/mTOR/IRF5 pathway in the temporal lobe brain tissue of the cerebral cortex of the mice in each group, and panels B to F show statistical bar graphs of the corresponding protein expression levels. Each group contained 8 mice. Data are presented as $\bar{x}\pm s$, and comparisons of differences among multiple groups were performed by one-way ANOVA with Tukey's follow-up test. Compared with the SAMR1 mice as normal control, ^**^P < 0.01. Compared with the untreated SAMP8 accelerated aging mice, ▴ *P* < 0.05 and ▴▴ *P* < 0.01.

**Fig. 10:**
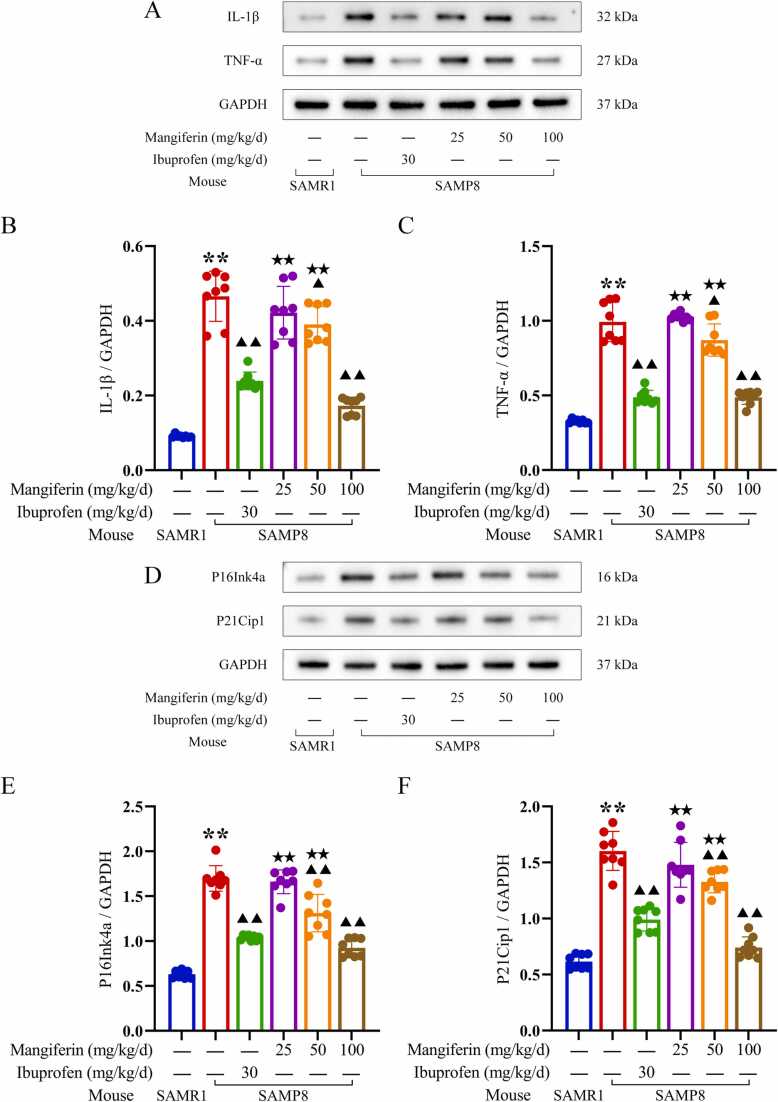
Inhibitory effects of mangiferin on neuroinflammation and neuronal cell senescence in SAMP8 accelerated aging mice. 8-week-old normal aging SAMR1 mice were used as normal control group. The 8-week-old SAMP8 mice in the remaining drug intervention groups were treated with ibuprofen or different doses of mangiferin for a period of 4 weeks. Panel A presents the Western blotting results of IL-1β and TNF-α proteins in the temporal lobe brain tissues of the cerebral cortex of the mice in each group, while panels B and C show the statistical bar graphs of IL-1β and TNF-α protein expression levels. Panel D provides the results of Western blotting of P16Ink4a and P21Cip1 proteins in temporal lobe brain tissues of the cerebral cortex of mice in each group, while panels E and F show the statistical bar graphs of P16Ink4a and P21Cip1 protein expression levels. Each group contained 8 mice. Data are presented as $\bar{x}\pm s$, and comparisons of differences among multiple groups were performed by one-way ANOVA with Tukey's follow-up test. Compared with the SAMR1 mice as normal control, ^**^P < 0.01. Compared with the untreated SAMP8 accelerated aging mice, ▴ *P* < 0.05 and ▴▴ *P* < 0.01. Compared with the SAMP8 accelerated aging mice treated with ibuprofen, ^★^*P* < 0.05 and ^★★^*P* < 0.01.

**Fig. 11:**
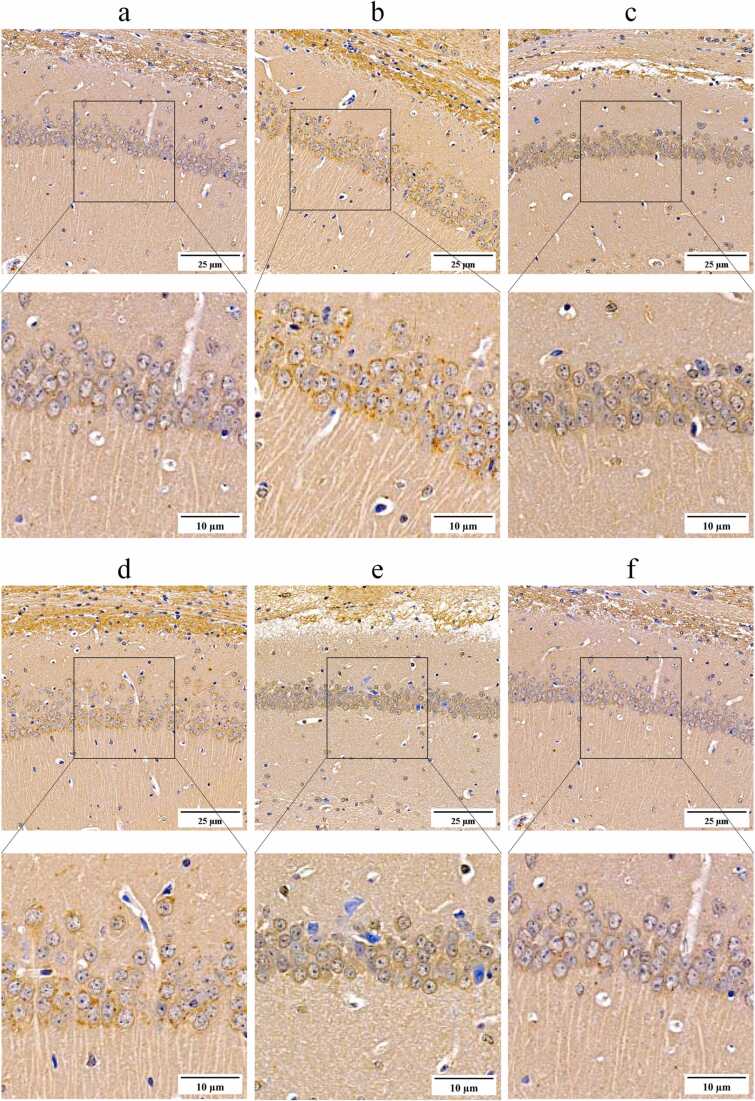
Immunohistochemical staining to observe the in-situ expression of P16Ink4a protein in brain tissues from the CA1 region of the mouse brain hippocampus. The target protein was sequentially labeled with a specific primary antibody and HRP-conjugated secondary antibody, followed by DAB color development. Positive staining manifests as brown or dark brown coloration. The content of the rectangular boxed area in each low magnification image is shown in the high magnification view below it. The experimental subjects included 8-week-old normal senescent SAMR1 mice (group a), 8-week-old SAMP8 mice not subjected to pharmacological interventions (group b), 8-week-old SAMP8 mice treated with 30 mg/kg/d of ibuprofen (group c), 8-week-old SAMP8 mice treated with 25 mg/kg/d of mangiferin (group d), 8-week-old SAMP8 mice treated with 50 mg/kg/d of mangiferin (group e), and 8-week-old SAMP8 mice treated with 100 mg/kg/d of mangiferin (group f). The duration of the drug treatment was 4 weeks.

**Fig. 12:**
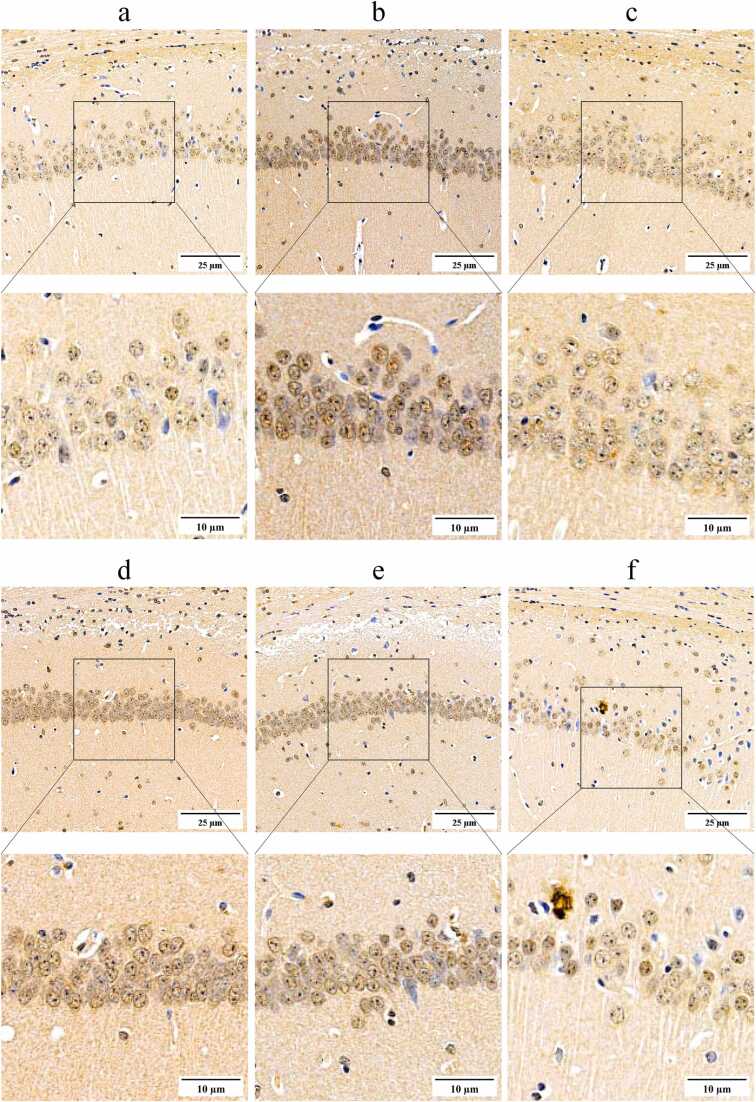
Immunohistochemical staining to observe the in-situ expression of P21Cip1 protein in brain tissues from the CA1 region of the mouse brain hippocampus. The target protein was sequentially labeled with a specific primary antibody and HRP-conjugated secondary antibody, followed by DAB color development. Positive staining manifests as brown or dark brown coloration. The content of the rectangular boxed area in each low magnification image is shown in the high magnification view below it. The experimental subjects included 8-week-old normal senescent SAMR1 mice (group a), 8-week-old SAMP8 mice not subjected to pharmacological interventions (group b), 8-week-old SAMP8 mice treated with 30 mg/kg/d of ibuprofen (group c), 8-week-old SAMP8 mice treated with 25 mg/kg/d of mangiferin (group d), 8-week-old SAMP8 mice treated with 50 mg/kg/d of mangiferin (group e), and 8-week-old SAMP8 mice treated with 100 mg/kg/d of mangiferin (group f). The duration of the drug treatment was 4 weeks.

**Fig. 13:**
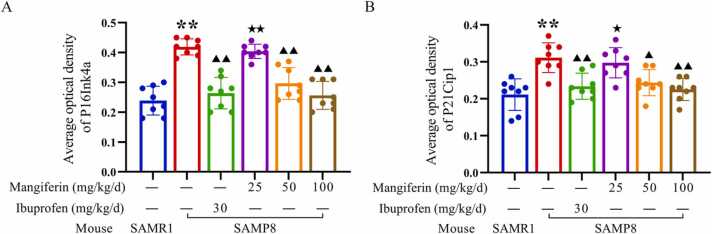
Comparison of the mean optical density of immunohistochemical staining of brain tissues of P16Ink4a (A) and P21Cip1 (B) in the hippocampal CA1 region of mice in each group. 8-week-old normal aging SAMR1 mice were used as the normal control group. The 8-week-old SAMP8 mice in the remaining drug intervention group were treated with ibuprofen or different doses of mangiferin for a period of 4 weeks. Data are expressed as $\bar{x}\pm s$ using one-way ANOVA and Tukey follow-up test. Compared with the SAMR1 mice as normal control, ^**^P < 0.01. Compared with untreated SAMP8 accelerated aging mice, ▴ *P* < 0.05 and ▴▴ *P* < 0.01. Compared with SAMP8 accelerated aging mice treated with ibuprofen, ^★^*P* < 0.05 and ^★★^*P* < 0.01.

As demonstrated in Fig. 7, Fig. 8, immunoblot analysis revealed elevated levels of the inflammatory cytokines IL-1β (0.47 ± 0.07) and TNF-α (0.99 ± 0.13) in the brain tissues of untreated SAMP8 mice. In comparison to the levels of IL-1β (0.09 ± 0.00) and TNF-α (0.33 ± 0.01) observed in the brain tissues of SAMR1 mice, which served as normal controls, the untreated SAMP8 mice exhibited significantly elevated levels of these inflammatory cytokines (*P* < 0.01). These findings provide relevant evidence to support the hypothesis that neuroinflammation is a prominent feature of SAMP8 mice. There were also lower levels of Phospho-(AMPKα1 +AMPKα2) (0.32 ± 0.03) in the brain tissue of SAMP8 mice, while higher levels of phospho-mTOR (S2448) (0.18 ± 0.02) and IRF5 (0.87 ± 0.08) were also present in the brain tissue of SAMR8 mice, which were similar to the higher levels of Phospho-(AMPKα1 +AMPKα2) (0.89 ± 0.09) and the lower levels of phospho-mTOR (S2448) in the brain tissue of SAMR1 mice(0.09 ± 0.01) and IRF5 (0.25 ± 0.02) were significantly different (*P* < 0.01). These findings suggest that there is a significant neuroinflammation associated with the AMPK-mTOR-IRF5 pathway in the brain tissue of SAMP8 mice. Neuroinflammation in SAMP8 mice was accompanied by abnormalities in markers associated with cell senescence in brain tissues, with higher levels of P16Ink4a (1.67 ± 0.14) and P21Cip1 (1.60 ± 0.17), significantly higher than levels of P16Ink4a (0.63 ± 0.04) and P21Cip1 (0.61 ± 0.06) in SAMR1 mice as normal controls (*P* < 0.01). In addition, immunohistochemical staining analyses of the hippocampal region showed similar alterations (Figs. 9–13), with higher levels of P16Ink4a (0.42 ± 0.03), and P21Cip1 (0.31 ± 0.04) positively stained optical density visible in the brain tissue of untreated SAMP8 mice, whereas in SAMR1 mice, the levels of P16Ink4a (0.24 ± 0.05), and P21Cip1 (0.21 ± 0.04) positive staining optical density levels were significantly lower than the former (*P* < 0.01). These findings support the idea that neuroinflammation accelerates neuronal cell senescence.

The immunoblot analysis revealed a distinct profile for each drug intervention group in terms of the regulation of the AMPK/mTOR/IRF5 pathway (Fig. 9). In comparison with untreated SAMP8 mice, brain tissues of SAMP8 mice treated with 100 mg/kg/d mangiferin exhibited elevated Phospho-(AMPKα1 +AMPKα2) (0.80 ± 0.14) levels (*P* < 0.01), while phospho-mTOR (S2448) (0.10 ± 0.01) and IRF5 (0.36 ± 0.05) levels were reduced (*P* < 0.01). Furthermore, analysis of brain tissues from SAMP8 mice treated with 50 mg/kg/d mangiferin revealed higher levels of Phospho-(AMPKα1 +AMPKα2) (0.68 ± 0.08) and lower levels of IRF5 (0.70 ± 0.08), which were statistically different from those observed in untreated SAMP8 mice (*P* < 0.01), but phospho-mTOR (S2448) (0.21 ± 0.02) levels did not show statistical differences, although this dose of mangiferin also demonstrated significant inhibition of neuroinflammation and delayed neuronal cell senescence in a favourable manner. Conversely, the brain tissues of SAMP8 mice treated with 30 mg/kg/d ibuprofen exhibited comparable levels of Phospho-(AMPKα1 +AMPKα2) (0.39 ± 0.03), phospho-mTOR (S2448) (0.15 ± 0.01), and IRF5 (0.79 ± 0.08) to those observed in untreated SAMP8 mice. These differential results suggest that the pharmacological effects of mangiferin in suppressing neuroinflammation and delaying neuronal cell senescence are closely related to the modulation of the AMPK/mTOR/IRF5 pathway, whereas the mechanism of action of ibuprofen, which produces similar pharmacological effects, is not.

Following a period of 4 weeks during which SAMP8 mice were administrated 30 mg/kg/d ibuprofen or 50, 100 mg/kg/d mangiferin, immunoblot analysis revealed that brain tissues exhibited reduced levels of IL-1β (0.24 ± 0.02, 0.39 ± 0.05 and 0.17 ± 0. 02, respectively) and TNF-α (0.49 ± 0.05, 0.87 ± 0.11 and 0.49 ± 0.05, respectively) were lower than in untreated SAMP8 mice, and the levels of P16Ink4a (1.04 ± 0.03, 1.31 ± 0.21, and 0.92 ± 0.10, respectively), and P21Cip1 (0.99 ± 0.10, 1.33 ± 0.10, and 0.74 ± 0.09, respectively) were lower, which were statistically significant (*P* < 0.05 or 0.01) (Fig. 10). Immunohistochemical staining analyses of the hippocampal region showed similar changes (Fig. 11, Fig. 12), with SAMP8 mice in these three treatment groups having P16Ink4a (0.27 ± 0.05, 0.29 ± 0.06 and 0.26 ± 0.05, respectively), P21Cip1 (0.23 ± 0.04, 0.24 ± 0.04 and 0.22 ± 0.03, respectively) positive staining optical density levels were lower than in untreated SAMP8 mice, and the difference was statistically significant (*P* < 0.05 or 0.01) (Fig. 9, Fig. 10). This study demonstrated that the administration of 100 mg/kg/d mangiferin exhibited comparable efficacy in the down-regulation of brain tissue IL-1β, TNF-α, P16Ink4a and P21Cip1 levels to that of 30 mg/kg/d ibuprofen. Furthermore, both doses were found to be more effective than 50 mg/kg/d mangiferin (Fig. 10). However, 25 mg/kg/d mangiferin was not found to be effective in inhibiting neuroinflammation or delaying neuronal cell senescence. These findings suggest that appropriate doses of both ibuprofen and mangiferin are capable of inhibiting neuroinflammation and thereby delaying neuronal cell senescence.

## 4 Discussion

The onset of AD and other neurodegenerative disorders is closely linked to neuroinflammation, a process that is often self-amplifying and self-sustaining, exacerbated by specific pathological factors such as the abnormal aggregation of Aβ (Zucca et al., 2023, Liu et al., 2024). The establishment of a chronic neuroinflammatory microenvironment can accelerate neuronal cell senescence and cognitive decline (White et al., 2017, Tao et al., 2018). In this study, we simulated the neuroinflammatory microenvironment *in vitro* by inducing microglial activation with Aβ, thereby recreating the inflammation-induced accelerated neuronal cell senescence. Consistent with previous findings, our results revealed significant neuroinflammation and neuronal cell senescence in the brain tissue of SAMP8 accelerated aging mice, indicating that microglial activation can indeed generate a neuroinflammatory microenvironment that accelerates neuronal cell senescence (Salminen et al., 2009). Consequently, inhibiting microglial activation may represent a potential therapeutic target for treating neurodegenerative diseases such as AD. Furthermore, this study elucidated that the AMPK/mTOR/IRF5 pathway is a critical mechanism underlying Aβ-mediated microglial activation. Both *in vitro* and *in vivo* experiments demonstrated that Aβ inhibits the phosphorylation of AMPK in microglia, thereby impeding its negative regulation of mTOR (Jung et al., 2022). The decrease in phosphorylated AMPK ultimately leads to an upregulation of IRF5 expression, promoting the differentiation of microglia into a pro-inflammatory phenotype. This phenotypic shift results in the enhanced secretion of pro-inflammatory cytokines, chemokines, and growth factors by microglia (Zhu et al., 2016). These factors collectively contribute to the establishment and maintenance of a chronic neuroinflammatory microenvironment, which ultimately accelerates neuronal cell senescence (Fig. 10). Based on these findings, pharmacological agents that can modulate the AMPK/mTOR/IRF5 signaling pathway may inhibit microglial activation, thereby offering significant therapeutic potential for alleviating neuroinflammation and neuronal cell senescence in patients with neurodegenerative diseases, such as AD.

**Fig. 14:**
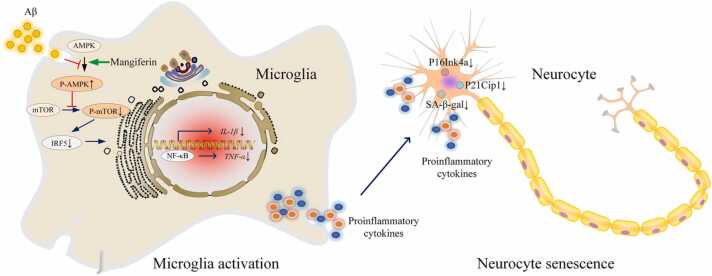
Schematic mechanism of action of natural polyphenol mangiferin in delaying neuronal cell senescence by inhibiting microglia activation-mediated neuroinflammation. Aβ activates microglia through the AMPK-mTOR-IRF5 pathway, which subsequently induces neuroinflammation and accelerates neuronal cell senescence. The natural polyphenol mangiferin has been shown to activate AMPK in microglia, inhibit mTOR activation, and downregulate IRF5 expression, effectively preventing microglial activation. These alterations lead to a reduction in neuroinflammation, thereby postponing the onset of neurocyte senescence.

Natural products are increasingly recognized as valuable resources for mitigating the progression of neurodegenerative diseases, such as AD (Tzvetkov and Atanasov, 2018). Recent discoveries of numerous bioactive compounds from diverse sources, including plants, microbes, and marine organisms, have demonstrated promising pharmacological activities against AD (Hamsalakshmi et al., 2022, Sharma et al., 2023, Zhang et al., 2023). Among these naturally derived compounds, mangiferin has garnered significant attention due to its remarkable anti-inflammatory properties, as evidenced by multiple studies (Liu et al., 2021, Qin et al., 2021). Our previous research has confirmed the anti-inflammatory effects of mangiferin, revealing its multi-target and multi-pathway mechanisms in inhibiting neuroinflammation (Wei et al., 2016, Wei et al., 2014). The findings of this study indicate that mangiferin significantly alleviates neuroinflammation and delays neuronal cell senescence, with high-dose mangiferin exhibiting slightly superior efficacy compared to ibuprofen, a commonly used anti-inflammatory drug, which may benefit patients suffering from neurodegenerative diseases like AD. Regarding long-term medication safety, ibuprofen is not an ideal choice (Nurmohamed, 2017), whereas mangiferin demonstrates favorable drug safety profiles (Villas Boas et al., 2019, Mirza et al., 2021). Importantly, these dose-dependent effects are mediated through the activation of AMPK, which inhibits mTOR-mediated upregulation of IRF5 expression. As a key regulator of energy metabolism, AMPK activation has been associated with various beneficial biological effects, including improvements in glucose and lipid metabolism. Therefore, in addition to mitigating the neurotoxic effects of Aβ, mangiferin holds considerable promise for enhancing glucose and lipid metabolism disorders that are closely linked to neuroinflammation, a critical pathological mechanism underlying AD. These findings underscore the therapeutic potential of mangiferin in the treatment of neurodegenerative diseases, particularly AD. By inhibiting the mechanistic target of mTOR, mangiferin may facilitate neuronal autophagy, thereby enhancing the clearance of hyperphosphorylated tau proteins and preventing the formation of neurofibrillary tangles, which represent another critical pathological mechanism in AD (Cai et al., 2012). Additionally, the inflammatory microenvironment is intricately linked to neuronal cell senescence and plays a significant role in the pathological processes affecting various essential functional cells, including vascular endothelial cells. The anti-inflammatory properties of natural polyphenols, such as mangiferin, may provide valuable benefits in mitigating cell inflammaging (Huang et al., 2024, You et al., 2024). However, it is crucial to establish robust research evidence to confirm these promising effects. While mangiferin has demonstrated a significant capacity to inhibit neuroinflammation and associated neuronal aging, this alone does not suffice to assert that it will yield optimal outcomes in enhancing cognitive function or in delaying or reversing the progression of AD. Only direct evidence from clinical drug trials can provide the necessary evidential strength to support such conclusions.

Neuroinflammation and neuronal cell senescence are critical pathological factors contributing to the progressive decline in cognitive function among individuals with AD (Motta et al., 2023, Ribeiro, 2023). Drug trials targeting these factors are relatively common. Preliminary research indicates that long-term use of nonsteroidal anti-inflammatory drugs (NSAIDs) may help prevent attention deficits in certain populations (Zandi and Breitner, 2001). However, clinical trials assessing the efficacy of NSAIDs in treating AD have yielded inconsistent results, failing to demonstrate the anticipated clinical benefits (Alzheimer's Disease Cooperative Study, 2003, McGeer and McGeer, 2007). These contradictory findings underscore the necessity for further research and a more profound understanding of the mechanisms underlying AD to accurately evaluate the therapeutic potential of NSAIDs in its treatment. Given that AD is a multifactorial disease arising from the interplay of various pathological mechanisms, it is nigh impossible that single-target drugs will achieve satisfactory efficacy (Chauhan et al., 2022). The lack of success in clinical trials for NSAIDs and monoclonal antibody therapies targeting Aβ partially supports this perspective (Alzheimer's Disease Cooperative Study, 2003, Bapineuzumab 301 and 302 Clinical Trial Investigators, 2014, Kwon et al., 2015). In contrast to NSAIDs, mangiferin exerts its anti-inflammatory effects through multiple targets and pathways, setting it apart from NSAIDs. Mangiferin has been shown to inhibit oxidative stress (Arora et al., 2020), enhance cellular metabolism (Mei et al., 2023), and improve cellular resistance to damage (Wang et al., 2022), thereby mitigating neuroinflammation and neuronal cell senescence within the pathological framework of AD. Given that mangiferin operates through various pathways, exploring its therapeutic effects on AD appears to be a reasonable endeavor. A comprehensive understanding of the complex etiology of AD will facilitate the advancement of natural products like mangiferin, which exhibit multi-target and multi-pathway pharmacological actions, toward becoming effective therapeutic interventions (Koynova and Tenchov, 2018, Woon et al., 2022). Our research findings support the potential of mangiferin as a lead compound for the treatment of AD.

The use of anti-inflammatory drugs to mitigate neuroinflammation in the treatment of AD has been a subject of ongoing debate. Inconsistent results have emerged from both preclinical and clinical studies regarding the efficacy of these medications. For example, naproxen demonstrated a reduction in cognitive decline compared to placebo in patients experiencing slow cognitive decline; however, the opposite effect was observed in patients with rapid cognitive decline. These findings indicate that the timing of anti-inflammatory drug administration, along with the individual characteristics of patients, plays a critical role in determining treatment outcomes (Aisen et al., 2003). Glucocorticoids, recognized as potent anti-inflammatory agents, have been shown to significantly elevate levels of Aβ and promote tau protein phosphorylation in mouse models of AD (Green et al., 2006). Clinical trials have demonstrated that glucocorticoids are ineffective in treating AD (Aisen et al., 2000). The contradictory results regarding the efficacy of anti-inflammatory medications indicate that these drugs operate through complex and diverse mechanisms that remain to be fully understood. From a mechanistic standpoint, one potential explanation for the adverse effects associated with anti-inflammatory drugs is their pronounced suppression of microglial cell function, which is critical for the phagocytosis and clearance of Aβ plaques (Jantzen et al., 2002, Koenigsknecht and Landreth, 2004). In patients with late-stage AD, where Aβ plaque accumulation is already substantial, the inhibition of microglial activation by anti-inflammatory drugs undermines the capacity of glial cells to clear Aβ plaques and facilitates the release of soluble Aβ, thereby harming adjacent neurons (Davis, 2002). Therefore, careful patient selection, precise timing, and thorough disease monitoring are essential to harness the potential benefits of anti-inflammatory drugs in the treatment of AD (Jordan et al., 2020). In the early stages of AD, anti-inflammatory drugs may slow neuronal cell senescence by mitigating neuroinflammation, thereby improving cognitive decline in patients (Lleó et al., 2004). Thus, the inhibition of microglial activation with anti-inflammatory drugs prior to extensive Aβ plaque deposition may offer a promising approach to alleviate neuroinflammation and delay the progression of AD (d'Errico et al., 2022).

Significant knowledge gaps remain in our understanding of the pathogenesis of AD, highlighting the need for further investigation. The lack of suitable animal models that accurately replicate the complex pathogenesis of human AD severely hinders drug development (Banerjee et al., 2022). Existing animal models are inadequate in capturing the multifaceted nature of human AD, which poses substantial limitations on the evaluation of drug efficacy both *in vitro* and *in vivo* (Neta et al., 2022). Therefore, it is crucial to conduct thorough research on the pathogenesis of AD and to develop more appropriate animal models that can faithfully reproduce its underlying mechanisms. This effort is vital for the advancement of effective treatment strategies for AD (Trujillo-Estrada et al., 2022).

## 5 Conclusions

In conclusion, mangiferin has the ability to modulate the AMPK/mTOR/IRF5 pathway, effectively inhibiting microglial activation and alleviating neuroinflammation. These pharmacological actions result in a significant delay in neuronal cell senescence. This study provides valuable insights into the mechanisms through which mangiferin addresses neuroinflammation and neurodegenerative diseases.

## Funding

This study was made possible by the generous funding from the Guangxi Natural Science Foundation of China (General program, Grant No. 2020GXNSFAA297258) and the Guangxi First-class Discipline: Traditional Chinese Medicine (Scientific Research of Guangxi Education Department [2022] No. 1).

## CRediT authorship contribution statement

**Chi Rui:** Data curation. **Kong Wen:** Data curation. **Wei Zhiquan:** Writing – review & editing, Project administration, Methodology, Investigation, Funding acquisition, Conceptualization. **Yan Li:** Writing – review & editing, Validation, Supervision, Methodology, Investigation. **Chen Xiaoli:** Investigation, Formal analysis, Data curation. **Nong Wei:** Writing – original draft, Methodology, Investigation, Formal analysis, Data curation. **Feng Xueping:** Validation, Software. **Chen Yixin:** Software, Formal analysis, Data curation.

## Conflict of interest and Compliance with ethical standards

These authors of this work confirm that there are no potential conflicts of interest to be reported. It is confirmed that all authors have granted their consent for submission of this manuscript for publication. The animal experimental protocol employed in this study underwent thorough examination and received approval from the Ethics Committee of Guangxi University of Traditional Chinese Medicine (approval No. DW20211210–204). These authors certify that they were carried out in accordance with the National Institute of health Guide for the Care and Use of Laboratory Animals (NIH Publications No. 80–23) revised 1996.

## Declaration of Competing Interest

These authors of this work confirm that there are no potential conflicts of interest to be reported. It is confirmed that all authors have granted their consent for submission of this manuscript for publication. This article has not been previously published, nor has it been submitted for consideration to any other journal.
